# Effects of different morphological types of enlarged vestibular aqueduct on clinical onset and cochlear implantation

**DOI:** 10.3389/fmed.2026.1944240

**Published:** 2026-09-23

**Authors:** Peng Zhu, Kun Ni, Xiao Yan Li, Zheng Nong Chen

**Affiliations:** 1 Department of Otorhinolaryngology, Shanghai Jiao Tong University Affiliated Sixth People’s Hospital, School of Medicine, Shanghai Jiao Tong University, Shanghai, China; 2 Department of Otorhinolaryngology, Shanghai Children’s Hospital, School of Medicine, Shanghai Jiao Tong University, Shanghai, China

**Keywords:** clinical onset, cochlear implantation, different morphological features, enlarged vestibular aqueduct, speech rehabilitation outcomes

## Abstract

**Objective:**

To investigate the influence of different morphological features of the enlarged vestibular aqueduct (EVA) on clinical onset and postoperative speech rehabilitation outcomes in pediatric patients undergoing cochlear implantation (CI) at our institution.

**Methods:**

A retrospective analysis was conducted on children diagnosed with EVA who underwent primary CI at our hospital. Clinical data, audiological profiles, and imaging characteristics were reviewed. Patients were categorized based on hearing loss (HL) symmetry: symmetric HL group (interaural threshold difference < 20 dB) and asymmetric HL group (difference ≥ 20 dB). The symmetric HL group was further stratified by age of onset into early-onset and late-onset subgroups. Based on disease progression, patients were classified into sudden HL and fluctuating HL subgroups. Radiological measurements included the vestibular aqueduct (VA) aperture angle and length. Postoperative speech outcomes in the early-onset subgroup were followed up at 1–6 years post-CI and compared with a matched control group of children with normal inner ear structures undergoing CI during the same period.

**Results:**

Eighty-one children with EVA were enrolled. Fifty-six cases presented with symmetric HL, while 25 cases exhibited asymmetric HL. Regarding progression patterns, 24 cases were classified as fluctuating HL and 25 as sudden HL. In the symmetric HL group, the early-onset subgroup demonstrated significantly larger bilateral VA aperture angles and shorter bilateral VA lengths compared to the late-onset subgroup. In the asymmetric HL group, the ear with worse HL had a larger VA aperture angle but a shorter VA length than the better-hearing ear. Postoperative speech performance improved over time in both groups; however, the early-onset subgroup consistently scored lower across all four speech evaluation metrics at 1–6 years post-CI compared to the control group.

**Conclusion:**

A wide and shallow vestibular aqueduct appears to render children more susceptible to inner ear damage induced by intracranial pressure fluctuations. This morphological predisposition likely contributes to earlier onset, greater severity, and more sudden progression of hearing loss, ultimately compromising postoperative speech rehabilitation outcomes.

## Introduction

Enlarged Vestibular Aqueduct (EVA) is one of the most prevalent congenital inner ear malformations associated with sensorineural or mixed hearing loss. It was first identified and termed by Valvassori and Clemis in 1978 (1). Originating from the medial wall of the vestibule, the vestibular aqueduct typically follows an inverted J-shape. Under normal physiological conditions, its narrow and elongated structure serves to buffer the impact of intracranial pressure fluctuations on auditory function. Conversely, a dilated and dysmorphic vestibular aqueduct may transmit excessive intracranial pressure, thereby inducing inner ear damage.

Children with EVA exhibit marked clinical heterogeneity, characterized by variable degrees of hearing impairment and diverse ages of onset. Hearing loss may present as either bilateral symmetric or asymmetric decline, often manifesting as a fluctuating or progressive worsening of thresholds. Clinically, the morphology of the enlarged vestibular aqueduct is also highly variable. This study aims to elucidate the potential relationship between EVA morphological features and the diversity of clinical presentations. Additionally, we seek to evaluate the long-term impact of EVA on postoperative speech rehabilitation outcomes in affected children.

## Methods

This study retrospectively reviewed the clinical records, audiological data, and temporal bone CT scans of pediatric patients diagnosed with enlarged vestibular aqueduct (EVA) who underwent primary cochlear implantation (CI) at our institution between 2019 and 2025. Exclusion criteria included revision surgeries, syndromic hearing loss (e.g., Usher syndrome, Pendred syndrome), severe concomitant inner ear malformations (e.g., common cavity, IP-I), autism spectrum disorders, or other comorbidities that could interfere with postoperative speech development.

Patients were categorized into two groups based on their pure-tone average (PTA): a symmetric hearing loss group (interaural PTA difference < 20 dB) and an asymmetric hearing loss group (interaural PTA difference ≥ 20 dB).

1. Symmetric Hearing Loss Subgroup Analysis: Within the symmetric group, patients were further stratified by age of onset into an early-onset group and a late-onset group. Early-onset was defined as meeting the criteria for CI (hearing loss > 70 dB HL) within the first year of life. All other patients were classified as late-onset. Temporal bone CT measurements of the VA aperture angle and length were compared between these two subgroups to identify statistically significant differences.
2. Asymmetric Hearing Loss Subgroup Analysis: For patients in the asymmetric group, VA aperture angles and lengths (defined as the distance from the VA apex to the midpoint of the aperture) were measured separately for each ear. These parameters were then compared between the poorer-hearing ear and the better-hearing ear to assess for statistical significance.
3. Progression Pattern Analysis: Based on the reported clinical history, patients were classified into a sudden hearing loss group and a fluctuating hearing loss group. The triggers for hearing deterioration were analyzed. VA aperture angles and lengths were compared between these two groups to determine if morphological differences existed.
4. Postoperative Speech Outcome Assessment: The early-onset subgroup (with symmetric hearing loss) was followed up at 1, 2, 3, 4, 5, and 6 years postoperatively. A matched control group—comprising children who underwent CI during the same period, with no inner ear malformations, and matched for sex, age, and side of implantation—was established. Speech rehabilitation outcomes were evaluated using the Categories of Auditory Performance (CAP), Meaningful Use of Speech Scale (MUSS), Mandarin Intelligibility Test (MITS), and Speech Intelligibility Rating (SIR).

### Statistical analysis

Data distribution was assessed using the Shapiro–Wilk test. Variables with a *P*-value > 0.05 were considered to follow a normal distribution. Homogeneity of variance was evaluated using Levene's test (*P* > 0.05 indicated equal variances).

In the symmetric hearing loss group, both vestibular aqueduct (VA) aperture angles and lengths conformed to a normal distribution; therefore, Welch's independent samples t-test was employed for intergroup comparisons. For the asymmetric hearing loss group, VA aperture angles were normally distributed and analyzed using a paired t-test. However, VA lengths deviated from normality and were subsequently analyzed using the Wilcoxon signed-rank test.

Regarding the comparison between the fluctuating and sudden hearing loss groups: the left and right VA angles, as well as the left VA lengths in both groups, were normally distributed with homogeneous variances, allowing the use of an independent samples t-test. Conversely, the right VA lengths were normally distributed but exhibited heterogeneous variances; thus, Welch's *t*-test was applied.

Analysis of the precipitating factors between the fluctuating and sudden hearing loss groups was performed using the chi-square test (with simulated *P*-values based on 2,000 Monte Carlo simulations) and Fisher's exact test (also with simulated *P*-values, *B* = 2,000). Statistical analyses were conducted using SPSS Statistics version 24.0 (IBM Corp., Armonk, NY, USA). A two-tailed *P*-value < 0.05 was considered statistically significant.

## Results

Eighty-one children with Large Vestibular Aqueduct Syndrome (LVAS) were enrolled, with an age range of 7–179 months at initial visit. Temporal bone imaging revealed that incomplete partition type II (IP-II) malformations were highly prevalent, affecting 78 of 81 children (96.3%). The cohort comprised 50 males (61.7%) and 31 females (38.3%), showing no statistically significant gender difference in incidence (*P* > 0.05). Age distribution analysis revealed that 21 cases (25.9%; 18 males, 3 females) presented before 12 months of age, 10 cases (12.3%; 3 males, 7 females) between 1 and 2 years, 38 cases (46.9%; 22 males, 16 females) between 3 and 5 years, 10 cases (12.3%; 6 males, 4 females) between 6 and 11 years, and 2 cases (2.5%; 1 male, 1 female) between 12 and 17 years. Notably, the highest incidence occurred in the 3–5 year age group, followed by infants under one year; furthermore, males predominated within this infant subgroup.

Of the 81 patients, 56 (69.1%) exhibited symmetrical hearing loss (interaural threshold difference < 20 dB). This group was subdivided into an early-onset subgroup (*n* = 27, onset < 1 year) and a late-onset subgroup (*n* = 29, onset ≥ 1 year) (Figure 1; Table 1). Radiographic measurements demonstrated that the early-onset subgroup had significantly larger bilateral VA aperture angles compared to the late-onset subgroup (Left: 81.6° vs. 45.8°; Right: 79.2° vs. 44.2°, both *P* < 0.001). Conversely, the early-onset subgroup exhibited significantly shorter bilateral VA lengths (Left: 2.54 mm vs. 4.25 mm; Right: 2.50 mm vs. 4.78 mm, both *P* < 0.001) (Table 2).

**Figure 1:**
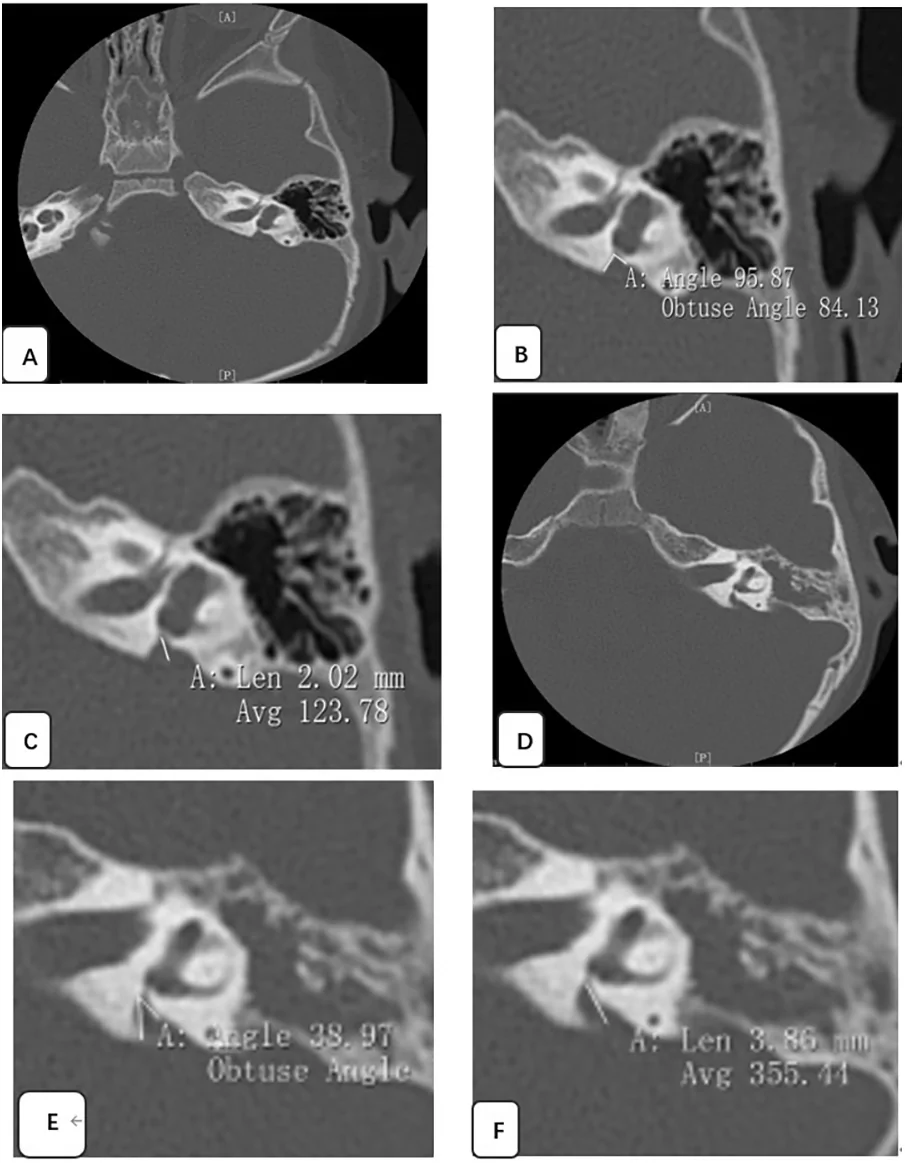
Comparison of clinical and imaging features between two symmetrical hearing loss patients. Early-Onset Case: Patient 1, a 9-month-old male, failed the newborn hearing screening. Auditory Brainstem Response (ABR) testing at 6 months of age revealed profound bilateral hearing loss (> 100 dB HL). High-resolution temporal bone CT demonstrated a markedly dilated vestibular aqueduct **(A)**. Radiographic measurements indicated a VA aperture angle of 95.87° **(B)** and a VA length of 2.02 mm **(C)**. Late-Onset Case: Patient 2, a 5-year-and-8-month-old male, passed the newborn hearing screening but presented with fluctuating hearing loss and recent regression in speech responsiveness. Preoperative audiometry confirmed profound bilateral hearing loss (> 100 dB HL). Temporal bone CT revealed a slender and elongated vestibular aqueduct **(D)**. Measurements showed a VA aperture angle of 38.97° **(E)** and a VA length of 3.86 mm **(F)**.

**Table 1:** Demographic grouping of enrolled children.

Group	Subgroup	Number of cases (n)	Percentage (%)
Total	—	**81**	**100**.**0**
Symmetrical hearing loss	Early-onset	27	33.3
	Late-onset	29	35.8
	**Subtotal**	**56**	**69**.**1**
Asymmetrical hearing loss	—	25	30.9

Symmetrical hearing loss was defined as an interaural difference in hearing thresholds of less than 20 dB. Conversely, asymmetrical hearing loss was defined as an interaural difference of 20 dB or greater. Based on the age at onset, the symmetrical hearing loss group was further stratified into an early-onset subgroup (age at onset < 1 year) and a late-onset subgroup (age at onset ≥ 1 year).

**Table 2:** Comparison of ipsilateral parameters between the early-onset and late-onset groups (welch's *t*-test).

Parameter	Early-onset group (*n* = 26) Mean ± SD	Late-onset group (*n* = 29) Mean ± SD	*t*-value	df	*P*-value
Left opening angle (°)	81.6 ± 11.4	45.8 ± 15.5	9.814	51.18	< 0.001
Right opening angle (°)	79.2 ± 12.9	44.2 ± 17.1	8.599	51.59	< 0.001
Left length (mm)*	2.54 ± 0.70	4.25 ± 0.86	−8.160	52.60	< 0.001
Right length (mm)*	2.50 ± 0.69	4.78 ± 1.03	−9.742	49.42	< 0.001

Twenty-five patients (30.9%) presented with asymmetrical hearing loss (interaural difference ≥ 20 dB) (Figure 2; Table 1). In these cases, the ear with poorer hearing showed a significantly wider VA aperture angle (65.22° vs. 43.79°, *P* < 0.001) (Figure 3) but a shorter VA length (3.50 mm vs. 4.26 mm, *P* < 0.001) compared to the better-hearing ear (Figure 4; Table 3). Serial audiometry of the better-hearing ears revealed a distinct high-frequency sloping configuration: average thresholds were 60 dB HL at 500 Hz, 60 dB HL at 1 kHz, 72.5 dB HL at 2 kHz, and 80 dB HL at 4 kHz. The threshold elevation at 4 kHz was statistically significant compared to lower frequencies (*P* < 0.05).

**Figure 2:**
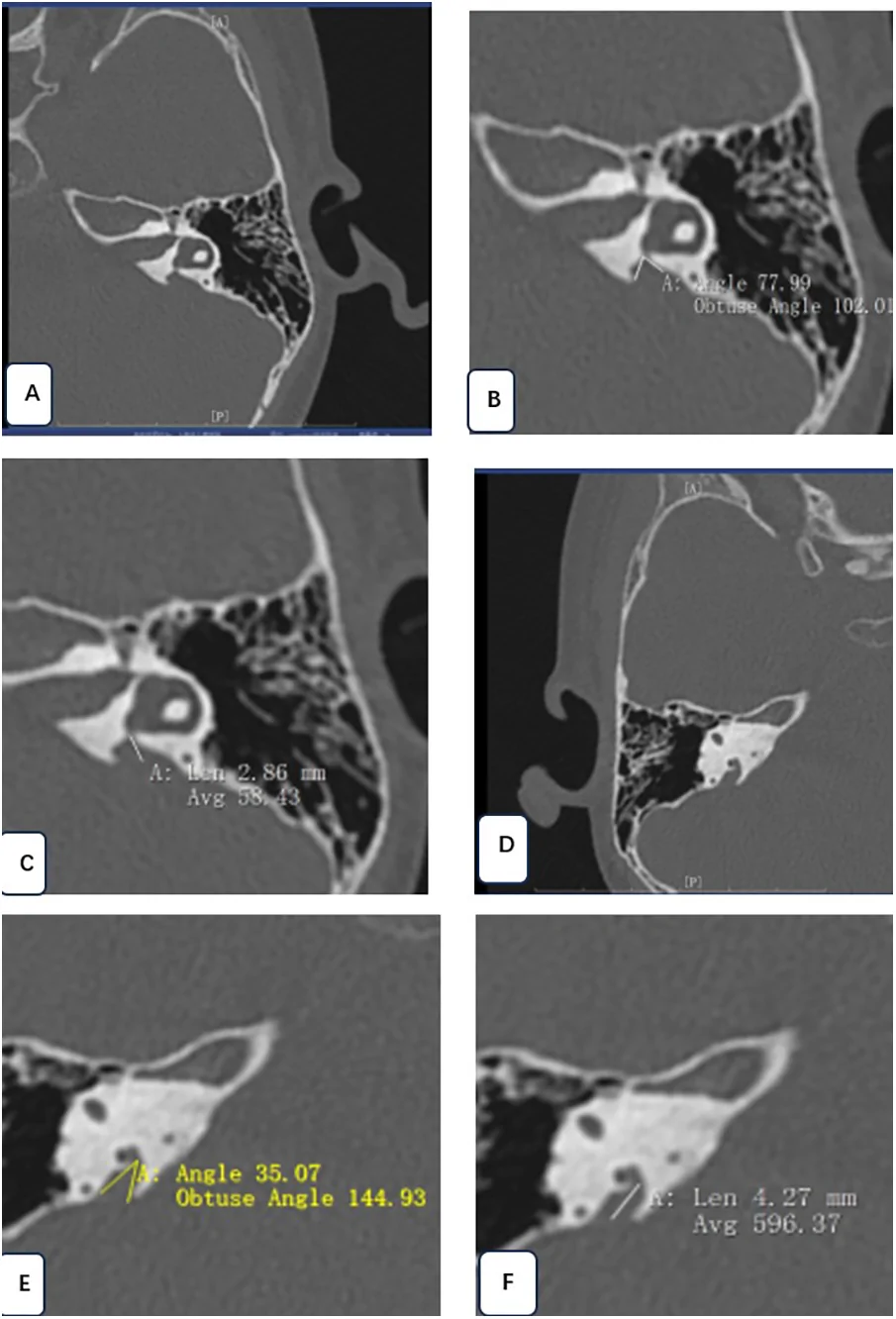
Comparison of clinical and imaging features between two sides of asymmetrical hearing loss patients. Case: Patient 3, a 4-year-and-11-month-old male, failed the newborn hearing screening. Initial ABR at 6 months showed moderate-to-severe bilateral loss. Recently, he experienced a progressive decline in hearing acuity in the left ear. Preoperative ABR indicated a threshold of 90 dB HL in the left ear and 60 dB HL in the right ear. Temporal bone CT demonstrated a widened left VA **(A)** with an aperture angle of 77.99° **(B)** and a length of 2.86 mm **(C)**. In contrast, the right VA appeared slender and elongated **(D)**, measuring 35.07° in aperture angle **(E)** and 4.27 mm in length **(F)**.

**Figure 3:**
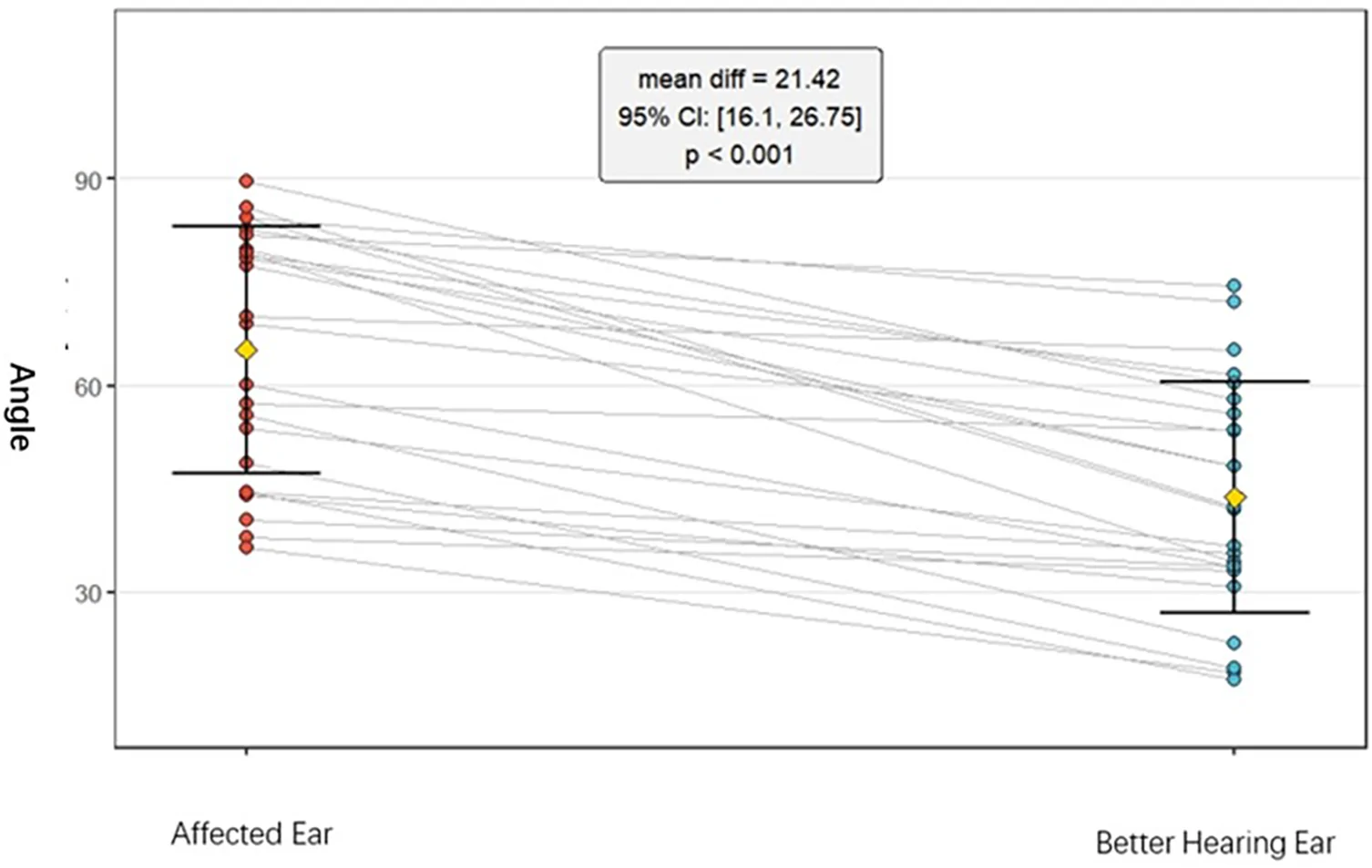
Shows that the vestibular aqueduct angle on the affected side was significantly larger than that on the better hearing side (*p* < 0.001).

**Figure 4:**
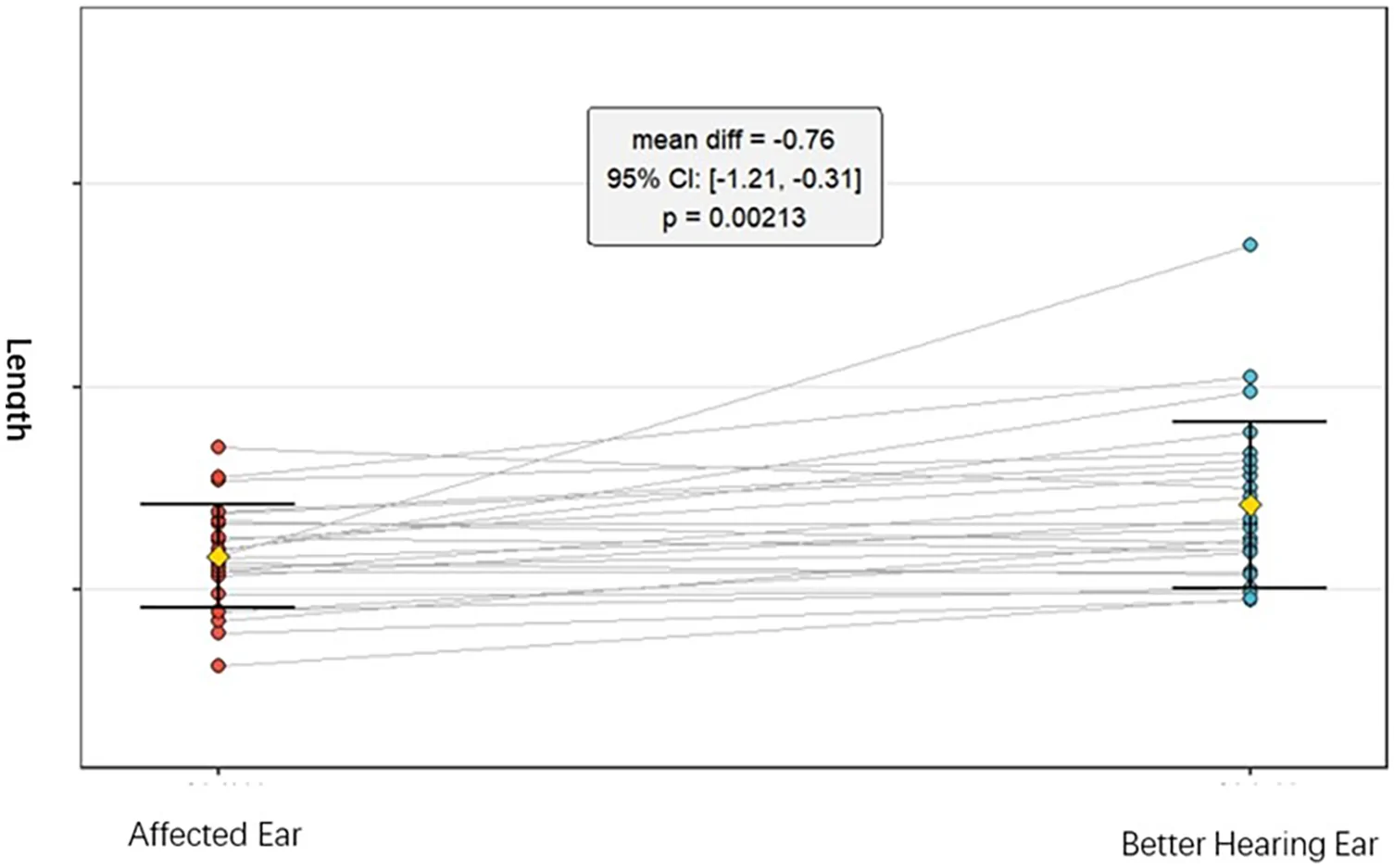
Shows that the vestibular aqueduct length on the affected side was significantly shorter than that on the better hearing side (*p* = 0.002).

**Table 3:** Comparison of parameters between the poorer-hearing and better-hearing sides (*n* = 24).

Variable	Side	Mean	SD	Median	Min	Max	Test Method	Statistic	*P*-value
Angle (°)	Poorer-hearing side	65.22	17.90	69.46	36.40	89.67	Paired t-test	8.316	< 0.001
	Better-hearing side	43.79	16.80	42.21	17.27	74.50			
Length (mm)*	Poorer-hearing side	3.50	0.76	3.48	1.88	5.10	Paired t-test	−3.459	0.002
	Better-hearing side	4.26	1.24	3.95	2.84	8.11			

Based on progression patterns, 24 patients were classified into the fluctuating HL group and 25 into the sudden HL group. The remaining 32 candidates were excluded from group allocation due to their inability to provide accurate descriptions of the disease onset pattern (Table 4). The sudden HL group exhibited significantly larger bilateral VA aperture angles than the fluctuating HL group (Left: 67.56° vs. 43.61°; Right: 64.85° vs. 41.80°, *P* < 0.001), coupled with significantly shorter bilateral VA lengths (Left: 3.30 mm vs. 4.19 mm; Right: 3.40 mm vs. 4.57 mm, *P* < 0.05) (Table 5). Analysis of precipitating factors revealed stark differences between groups: trauma accounted for 52% of cases in the sudden HL group versus only 8.7% in the fluctuating HL group, while upper respiratory tract infections (“colds”) were reported in 39.1% of the fluctuating HL group versus 16% in the sudden HL group (Table 6).

**Table 4:** Onset patterns of hearing loss in enrolled children.

Onset pattern	Number of cases (n)	Percentage (%)
Fluctuating hearing loss	24	29.6
Sudden hearing loss	25	30.9
Undetermined onset pattern	32	39.5
Total	**81**	**100**.**0**

**Table 5:** Descriptive statistics and comparative analysis of parameters between the fluctuating and sudden types.

Type	Variable	*N*	Mean ± SD	Median (Q1, Q3)	Range (Min–Max)	Homogeneity of Variance (P)	Test Method	Statistic	*P*-value
Fluctuating	Left angle (°)	23	43.61 ± 13.70	46.97 (35.35, 51.58)	9.83–71.68	0.154	Student's t-test	−4.705	< 0.001
Sudden		25	67.56 ± 20.60	65.01 (55.66, 77.39)	33.05–120.47				
Fluctuating	Right angle (°)	23	41.80 ± 16.44	43.70 (32.06, 56.48)	17.04–74.74	0.699	Student's t-test	−4.706	< 0.001
Sudden		25	64.85 ± 17.39	68.97 (51.60, 74.55)	34.51–103.24				
Fluctuating	Left length (mm)*	23	4.19 ± 0.98	4.01 (3.35, 4.93)	2.55–5.93	0.523	Student's t-test	3.283	0.002
Sudden		25	3.30 ± 0.91	3.38 (2.73, 3.96)	1.51–5.12				
Fluctuating	Right length (mm)*	23	4.57 ± 1.73	4.14 (3.32, 5.72)	1.60–9.16	0.010	Welch's *t*-test	2.906	0.007
Sudden		25	3.40 ± 0.90	3.45 (3.01, 3.80)	1.51–5.12				

**Table 6:** Etiology composition of the fluctuating and sudden types [*n* (%)].

Type	Unknown	Common Cold	Trauma	*χ* ^2^	*P*-value (Approx.)	Fisher's Exact *P*-value
Fluctuating (*n* = 23)	12 (52.2%)	9 (39.1%)	2 (8.7%)	10.725	0.0045	0.0060
Sudden (*n* = 25)	8 (32.0%)	4 (16.0%)	13 (52.0%)			

A total of 27 children were enrolled in the early-onset group, all of whom presented with an onset age and cochlear implantation age of less than 1 year (mean implantation age: approximately 10 months). Of these, 20 underwent bilateral implantation and 7 underwent unilateral implantation. To control for confounding variables, 27 children without inner ear malformations were matched for age, sex, and laterality of implantation. In the control group, the mean age at implantation was also approximately 10 months, with 21 cases of bilateral implantation and 6 cases of unilateral implantation. Longitudinal assessment of speech outcomes showed improvement across all four evaluation metrics over time in both groups. However, the early-onset subgroup consistently achieved significantly lower scores compared to the matched control group throughout the 1–6 year follow-up period (Figure 5). Specifically, parental reports indicated less distinct articulation at 3–4 years post-implantation, evolving into poorer linguistic organization and expressive skills by 5–6 years post-implantation in the EVA cohort compared to controls.

**Figure 5:**
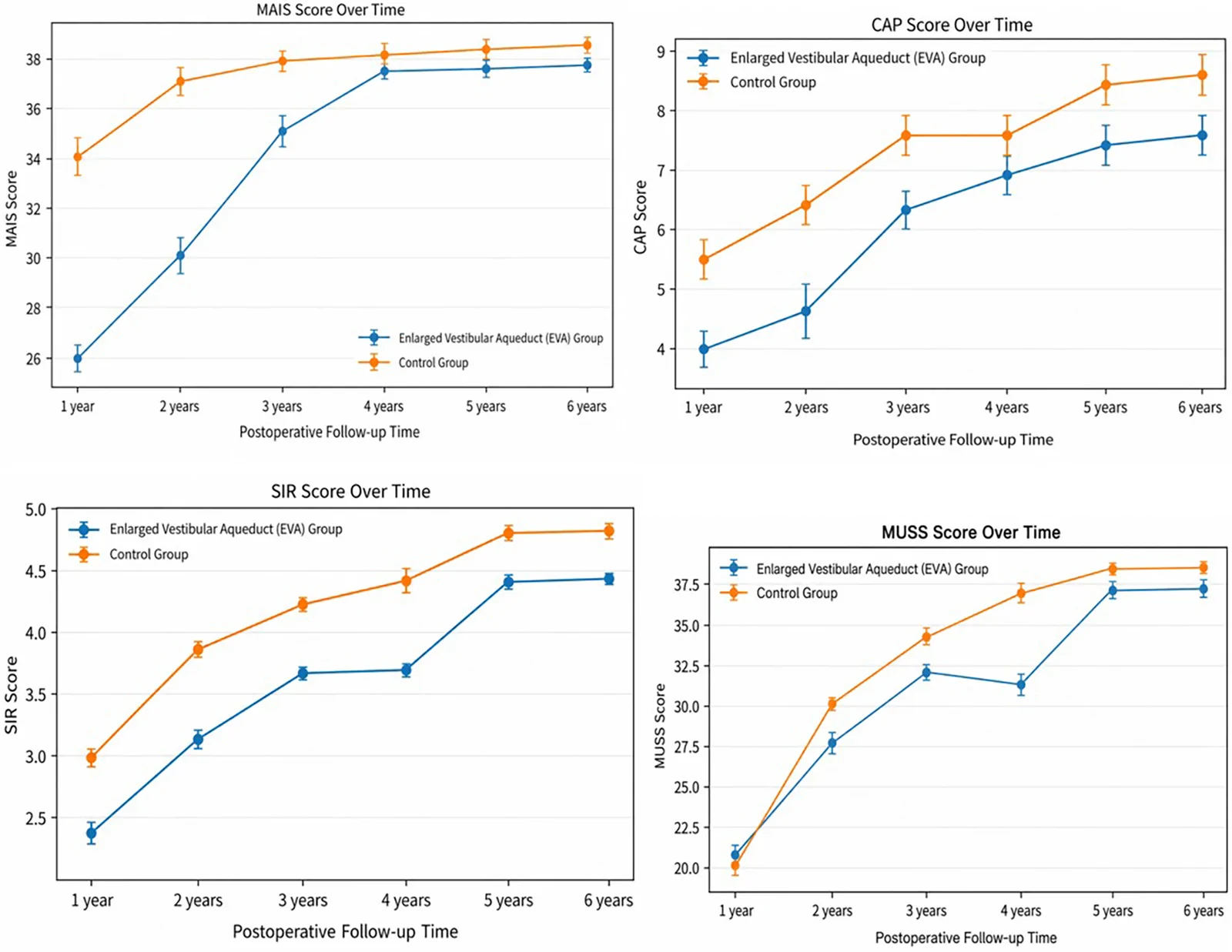
Serial assessments were conducted annually from 1 to 6 years post-implantation using the categories of auditory performance (CAP), speech intelligibility rating (SIR), mandarin speech perception test (MITS), and meaningful Use of speech scale (MUSS). Both the early-onset enlarged vestibular aqueduct (EVA) subgroup and the matched control group demonstrated significant overall improvement across all four metrics over time. However, the early-onset EVA subgroup consistently achieved significantly lower scores compared to the control group at every annual interval (all *P* < 0.05).

## Discussion

The vestibular aqueduct (VA) is a bony canal with a characteristic inverted “J” shape, originating from the medial wall of the vestibule and extending posterolaterally to open onto the posterior surface of the petrous temporal bone. It serves as the conduit between the vestibule and the endolymphatic sac, through which the venous drainage from the semicircular canals and utricle traverses en route to the sigmoid sinus (2–4).

The pathogenesis of EVA is widely attributed to a developmental arrest of the inner ear occurring around the seventh week of gestation. Embryologically, the VA is initially short, straight, and relatively wide; it gradually elongates and assumes its definitive inverted J-shape with maturation. Insults during early embryogenesis—such as maternal viral infections or ototoxic drug exposure—may impede this remodeling process, resulting in the persistence of a fetal-like, dilated morphology. Fundamentally, EVA is characterized by the enlargement of the endolymphatic sac and duct (5).

Clinically, EVA manifests with considerable heterogeneity. While some patients present with early-onset hearing loss, others exhibit a slow, fluctuating progression. Precipitating factors often include head trauma or upper respiratory tract infections. Hearing loss may be bilateral symmetric or asymmetric. Current research into the pathophysiology of EVA has predominantly focused on the genetic mutations of *SLC26A4* and the functional consequences of its encoded protein, pendrin (6, 7). While non-molecular mechanisms have been proposed to explain the phenotypic variability, and integrative models combining molecular and anatomical perspectives are emerging, the full spectrum of clinical manifestations cannot be solely explained by existing theories.

Two principal mechanistic hypotheses dominate the understanding of hearing loss in EVA. First, the “**pressure buffering hypothesis”** posits that unlike the normal VA which dampens intracranial pressure (ICP) fluctuations, a dilated VA allows direct hydraulic transmission of ICP to the membranous labyrinth, resulting in cochlear hair cell damage. Second, the “**endolymphatic reflux hypothesis”** suggests that the endolymphatic sac contains a hyperosmolar fluid (protein concentration higher than perilymph). Under elevated ICP, this hyperosmolar fluid may reflux through the enlarged VA into the basal turn of the cochlea via the dilated endolymphatic sac/duct, disrupting the physiology of the neurosensory epithelium (8).

Supporting the role of VA morphology in functional outcomes, Spencer et al. demonstrated a linear correlation between hearing loss severity and the mid-point diameter of the VA, underscoring hemodynamic pressure changes as a key pathogenic driver (9). Archibald et al. further corroborated that larger mid-point diameters correlate with poorer pure-tone averages and speech discrimination scores. Longitudinal data from Ascha et al. revealed that a 1.5 mm increase in the VA mid-point diameter was associated with a 21% decrease in speech recognition and a 17.5 dB elevation in speech reception thresholds over six years (9). Consequently, assessing VA morphology—particularly the mid-point diameter—is crucial when evaluating postoperative CI outcomes.

Our findings align with these observations. We noted that patients with early-onset and sudden hearing loss tended to have wider aperture angles and shorter VA lengths. This morphological profile likely correlates with a diminished capacity for pressure buffering and enhanced reflux potential, leading to accelerated hair cell and spiral ganglion cell (SGC) degeneration. Given that CI functions by electrically stimulating residual SGCs and viable hair cells, a greater loss of these neural elements—presumably more pronounced in cases with significant VA dilation—will inevitably compromise postoperative auditory rehabilitation. Therefore, the timing of hearing loss onset, pre-operative hearing levels, and specific VA morphological parameters (such as the mid-point diameter, aperture angle, and length) serve as vital prognostic indicators for CI success (10, 11).

Prior to hearing deterioration, children with EVA frequently report antecedent events such as upper respiratory tract infections (URTIs), febrile episodes, or head trauma—all of which elevate intracranial pressure (ICP). Mild ICP fluctuations induced by coughing during a cold may cause transient inner ear damage, manifesting as reversible fluctuating hearing loss. In contrast, traumatic events generate abrupt and forceful ICP surges, typically resulting in irreversible cochlear injury and sudden hearing loss. The pathophysiological mechanism likely parallels that of explosive blast injury to the cochlea. Our data support this analogy: the sudden hearing loss subgroup exhibited significantly wider VA aperture angles and shorter VA lengths. This morphology suggests that a wide and shallow vestibular aqueduct acts as a low-resistance conduit, facilitating the transmission of hydraulic pressure waves to the cochlea and precipitating sudden, irreversible damage.

Within the symmetrical hearing loss cohort, the early-onset subgroup displayed markedly wider apertures and shorter lengths compared to their late-onset counterparts. This finding implies that a wide and shallow configuration accelerates the timeline of cochlear insult. Similarly, in the asymmetrical hearing loss group, the ear with poorer thresholds possessed a wider and shallower VA than the better-hearing ear. This intrasubject comparison strongly suggests that, even under identical environmental stressors, morphological variants dictate the susceptibility to hearing loss, reinforcing the concept that the structural deformity itself is a primary driver of auditory pathology. Furthermore, preoperative ASSR profiling of the better-hearing ears revealed a distinct high-frequency sloping audiogram, with thresholds at 4 kHz deteriorating significantly more than at lower frequencies. This pattern mirrors the classic configuration seen in noise-induced or blast-related hearing loss, suggesting that early-stage EVA-related damage preferentially targets the basal turn of the cochlea.

Collectively, our results provide robust validation for the pressure buffering hypothesis. Crucially, this study is the first to systematically delineate the quantitative relationship between specific VA morphological parameters (aperture angle and length) and the phenotypic diversity of clinical onset. A wide and shallow aqueduct appears to lower the threshold for cerebrospinal fluid pressure waves to traumatize the cochlea, leading to earlier onset, greater severity, and sudden progression of hearing loss. These findings carry significant implications for emerging therapies. Even as gene therapy targeting *SLC26A4* gains traction, it may not fully mitigate hearing loss in these patients—much like how individuals with normal genetics can suffer from blast-induced deafness. Therefore, therapeutic strategies must extend beyond genetics to address the underlying osseous structural deformity. Future research should ideally shift focus to the embryonic stage, aiming to prevent the development of VA malformations altogether. For children already born with EVA, clinical management should prioritize minimizing the impact of ICP fluctuations to delay onset and preserve residual hearing. We intend to continue longitudinal follow-up to compare speech rehabilitation outcomes across different age groups and VA morphologies, further refining the diagnostic and therapeutic paradigms for this complex condition.

## Conclusion

Inner ear structural malformations represent a pivotal factor underlying hearing deterioration in children with enlarged vestibular aqueduct (EVA). A wide and shallow vestibular aqueduct likely renders cochlear hair cells more vulnerable to damage induced by intracranial pressure fluctuations, thereby predisposing affected children to earlier onset, greater severity, and more abrupt progression of hearing loss.

Furthermore, the morphologically enlarged aqueduct may exacerbate the loss of cochlear hair cells, which consequently compromises postoperative speech rehabilitation outcomes.

## Data Availability

The raw data supporting the conclusions of this article will be made available by the authors, without undue reservation.
